# Repeated exposure rivals lightweight training in Bayesian inference: accuracy, retention, and generalization

**DOI:** 10.3389/fpsyg.2026.1908176

**Published:** 2026-08-25

**Authors:** S. Pighin, P. Ghin, K. Tentori

**Affiliations:** Center for Mind/Brain Sciences, University of Trento, Trento, Italy

**Keywords:** Bayesian reasoning, dynamic visualization, natural frequencies, predictive values, training

## Abstract

Bayesian inference is fundamental to interpreting diagnostic evidence, yet individuals often fail to integrate base rates and test characteristics when evaluating test results. Key questions are whether these difficulties can be attenuated by brief, scalable interventions, whether possible improvements are maintained over time, and whether they generalize to different probability judgments. We addressed these questions in an online experiment involving 256 laypeople who were presented with medical-screening Bayesian problems in a natural-frequency format and asked to estimate either positive predictive value (PPV) or negative predictive value (NPV). Participants were assigned to one of three conditions: repeated exposure to the problems, brief text-based training based on highlighted wording, or brief dynamic training based on animated visualizations. They completed the task before and after the intervention, and again in a delayed post-test administered 8–10 days later, which also assessed generalization to the predictive value not practiced during the intervention (namely PPV following NPV and vice versa). Performance improved reliably from pre-test to immediate post-test and remained elevated at delayed post-test. Moreover, improvements generalized to the unpracticed predictive value. However, neither training condition conferred a consistent advantage over mere exposure to plain text. Accuracy did not vary by predictive value, with comparable performance across PPV and NPV, and error analyses suggested that incorrect responses were heterogeneous and only weakly captured by standard non-Bayesian categories. Together, these findings suggest that modest but significant gains in Bayesian reasoning can emerge from repeated task exposure, whereas highlighted text, dynamic visualization, and feedback do not confer additional benefit.

## 1 Introduction

Across a range of real-world contexts, most notably in medical diagnostics, individuals are required to update their beliefs in light of new evidence, such as test results or clinical indicators. Bayesian inference provides a normative benchmark for this process, specifying how prior probabilities should be revised when new information becomes available. However, decades of empirical research in cognitive science have consistently shown that even well-educated individuals, including healthcare professionals, often struggle to apply Bayesian principles correctly (Barbey and Sloman, 2007; Bar-Hillel, 1980; Hamm, 1996; Kahneman and Tversky, 1973). In particular, they tend to systematically misinterpret posterior probabilities, often failing to adequately incorporate base-rate information (e.g., Casscells et al., 1978; Bar-Hillel, 1980; Kahneman and Tversky, 1972; Tversky and Kahneman, 1974). This issue is not merely of theoretical interest but has clear practical implications. In medical settings, for example, failures in Bayesian reasoning can translate into flawed diagnoses, inappropriate treatments, and systematic distortions in risk communication (Doust, 2009; Gigerenzer and Edwards, 2003).

Despite the substantial body of research devoted to improving Bayesian reasoning, no single intervention has emerged as consistently effective across tasks, formats, and populations. Much previous work has focused on how numerical information is presented and processed. Several proposals have been advanced to simplify the numerical structure of Bayesian problems, for instance by replacing single-event probabilities with natural frequencies (e.g., Gigerenzer and Hoffrage, 1995; McDowell and Jacobs, 2017) or chances (e.g., Girotto and Gonzalez, 2001; Pighin et al., 2017), as well as by restructuring the phrasing of questions to better align with extensional reasoning (e.g., Girotto and Gonzalez, 2001; Pighin et al., 2015, 2018).

A parallel line of research has investigated whether traditional visual aids (e.g., icon arrays, tree diagrams, and Venn diagrams) make probabilistic relations among the relevant elements of Bayesian problems more transparent (e.g., Brase, 2009; Garcia-Retamero et al., 2015). Although some studies have reported performance gains, the overall evidence remains mixed: the effectiveness of static visualizations appears to depend critically on factors such as the specific task structure, numerical format, individual differences (e.g., in numeracy or spatial ability), and the way information is integrated with text (for a review, see Cui et al., 2023). The picture becomes even more nuanced when dynamic, rather than static, visual representations are considered. In principle, dynamic visualizations could reduce cognitive load, highlight the logical structure of information through signaling and motion, and support a more coherent integration of statistical concepts (Mayer, 2002); as such, they may be particularly well suited to representing nested sets, which are central to Bayesian inference. Accordingly, they have shown potential benefits in some domains, such as improving recall, facilitating the identification of probabilistic values, and supporting risk communication (e.g., Han et al., 2012; Zikmund-Fisher et al., 2012). However, their impact on probability computation and Bayesian inference remains largely unexplored.

A further strand of research suggests that Bayesian reasoning can be improved, at least to some extent, through targeted training. In particular, interventions that encourage participants to recode probabilistic information into more transparent representations, such as nested-set displays, have been shown to improve performance, with effects that, in some cases, persisted beyond the immediate training phase and even generalized to novel problems (e.g., Kurzenhäuser and Hoffrage, 2002; Sedlmeier and Gigerenzer, 2001). More recent experimental work has shown that tutorials employing visual strategies can yield modest performance gains (e.g., Starns et al., 2019), consistent with observational evidence of instructional programs combining multiple components (e.g., Büchter et al., 2022).

Crucially, however, the practical value of such aids depends on whether they can be delivered through feasible interventions. Existing training programs typically rely on explicit instruction, guided practice, or multiple components, which may limit their use when time and resources are scarce. Our first aim was therefore to compare the immediate and delayed effects of repeated task exposure with those of two brief trainings: a text-based intervention involving minimal highlighting of relevant wording and a dynamic intervention using animated visualizations.

Our second aim was to address a notable asymmetry in the literature. Specifically, most studies have examined positive predictive value (hereafter, PPV), the probability of having a condition given a positive test result (for a review, see Whiting et al., 2015), whereas comparatively little attention has been devoted to negative predictive value (hereafter, NPV), the probability of not having a condition given a negative test result (for exceptions, see Lyman and Balducci, 1993; Noguchi et al., 2002; Pighin and Tentori, 2021). This imbalance is clinically relevant because the two predictive values serve different clinical roles: whereas PPV may inform treatment decisions, NPV can help determine whether further diagnostic testing or monitoring is warranted. Although PPV and NPV involve analogous computations based on true positives and true negatives, respectively, this structural parallel does not imply that the two judgments are equally intuitive or cognitively accessible. Indeed, the relative contribution of the underlying parameters differs: PPV increases as prevalence rises, whereas NPV decreases; moreover, PPV is particularly influenced by specificity while NPV by sensitivity. These distinct relationships may affect how the relevant information is processed, potentially giving rise to different reasoning processes and patterns of bias susceptibility. Because this gap limits the generalizability of existing findings on how people interpret test results, we examined both PPV and NPV within the same framework to test whether the proposed interventions produced comparable immediate and delayed effects across the two judgments and whether these effects generalized from the practiced to the unpracticed predictive value.

## 2 Methods

We conducted an online experiment in which participants were asked to solve Bayesian inference problems with medical screening content. The experiment followed a 3 (training condition: no training, textual training, or dynamic training) x 2 (predictive-value condition: PPV vs. NPV) between-subjects design.

### 2.1 Participants

We recruited a sample of English-speaking participants residing in the United Kingdom through Prolific (Prolific Academic Ltd., London, United Kingdom), a commercial online participant-recruitment platform designed for scientific research (Palan and Schitter, 2018). Recruitment was restricted to UK residents to limit linguistic and contextual heterogeneity by drawing the sample from a single national context while retaining access to a large and diverse participant pool. Using Prolific’s prescreening filters, eligibility was limited to participants with an approval rate of at least 99% (i.e., fewer than 1% of their previous submissions on the platform had been rejected by researchers because of failed attention checks or other indications of inadequate responding), thereby increasing the expected reliability of the data. Participants were also required to access the study via a desktop or laptop computer to ensure that the stimuli, including the animations, were displayed as intended.

Of the participants who accessed the study, 61 discontinued before completing the pre-test phase (abandonment rate = 16.9%). Among the 300 participants who completed the pre-test phase, 44 did not proceed through all subsequent phases of the study (attrition rate = 14.7%) and were excluded from the analyses, resulting in a final sample of 256 participants: 137 females, 117 males, and 2 participants who did not report their gender. Participant age ranged from 19 to 77 years (M = 42.7, SD = 13.4). Educational attainment was distributed as follows: 14.8% held a high school diploma, 19.1% had attended college or university without completing a degree, 44.2% held a bachelor’s degree, and 21.9% held a master’s degree.

Participants received monetary compensation proportional to the time required to complete each phase (approximately £7.50/h). In addition, participants who completed all phases of the study received a £1 completion bonus to reduce attrition. Finally, to encourage engagement, a performance-based incentive of £1 was also offered to participants who correctly solved three problems randomly selected from those included in the task. The study was approved by the University of Trento’s Ethical Review Board, and all participants provided informed consent.

### 2.2 Materials and stimuli

**Figure 1:**
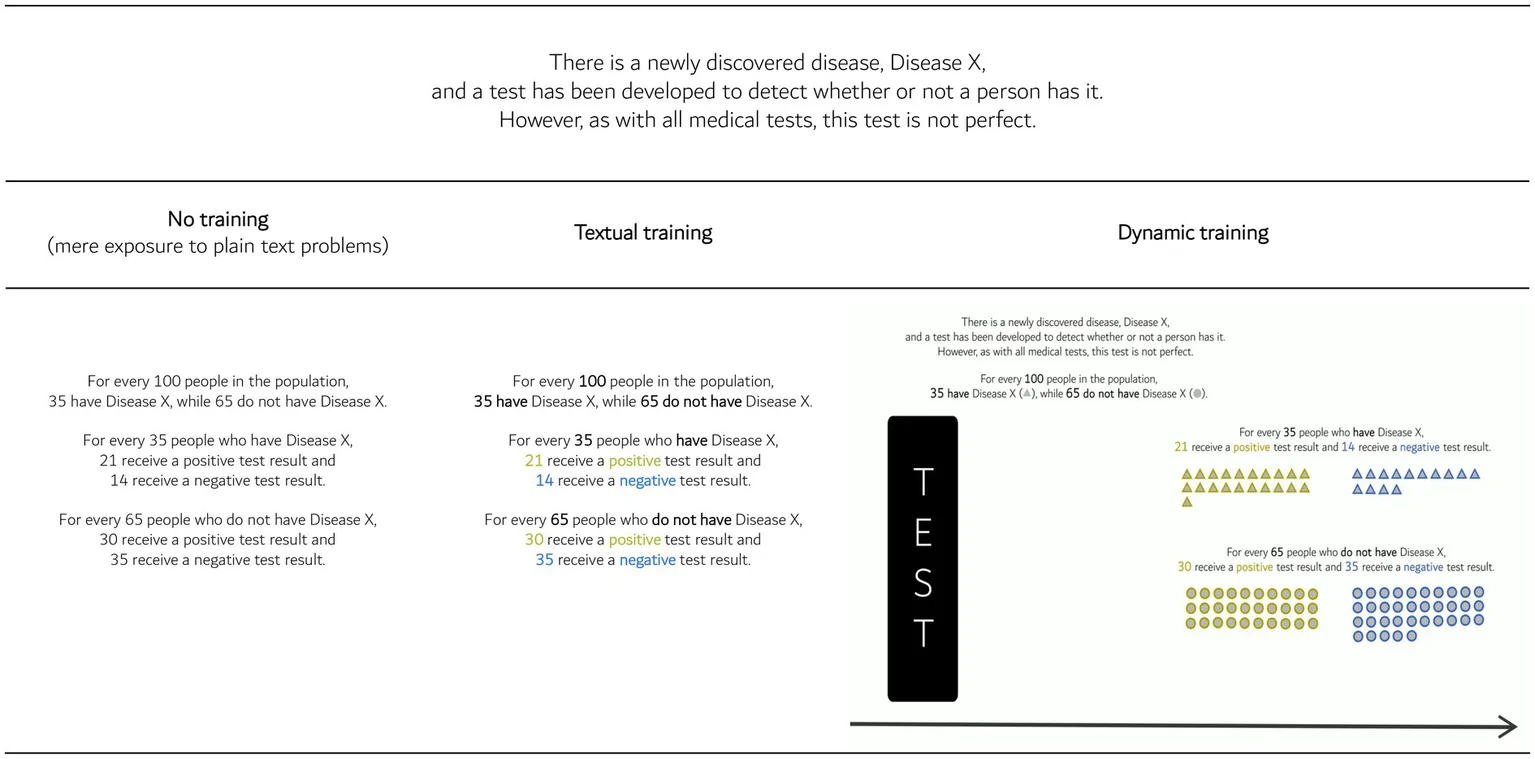
Example of the information provided in the Bayesian reasoning problems across the three experimental conditions: no training, text-based training, and dynamic training.

All experimental stimuli consisted of Bayesian reasoning problems framed within a medical screening scenario involving a fictitious “Disease X.” Each problem described a population of 100 individuals and specified the base rate, as well as the distribution of true positives, false positives, true negatives, and false negatives (see Figure 1). The full set of values used in the Bayesian problems is reported in Table 1. Importantly, all stimuli were constructed so that the computational complexity required to calculate the PPV and NPV was exactly the same (the exact wording of each question is reported below). Specifically, for each PPV problem, a corresponding NPV problem was created by reversing the prevalence of the disease while mirroring the underlying frequency structure. For example, consider Problems 1a and 1b. In both cases, the population consisted of 100 individuals, with 10 affected and 90 unaffected by Disease X in Problem 1a, and 90 affected and 10 unaffected by Disease X in Problem 1b. The true positives in Problem 1a were equal to the true negatives in Problem 1b, while the false positives in Problem 1a were equal to the false negatives in Problem 1b, yielding the same correct response (i.e., PPV_1a = NPV_1b = 47.06%). This allowed us to exclude the possibility that any differences in the estimation of PPV and NPV were due to differences in numerical difficulty or computational demands.

**Table 1:** Numerical values of the Bayesian inference problems used across experimental phases.

Phase	Problem	Predictive value	Prior probability	True positives	False negatives	True negatives	False positives	Correct response
Pre-test, immediate post-test, delayed post-test and generalization	1a	PPV	10	8	2	81	9	8/17
	1b	NPV	90	81	9	8	2	
	2a	PPV	8	5	3	70	22	5/27
	2b	NPV	92	70	22	5	3	
	3a	PPV	90	81	9	8	2	81/83
	3b	NPV	10	8	2	81	9	
	4a	PPV	92	70	22	5	3	70/73
	4b	NPV	8	5	3	70	22	
Training	5a	PPV	21	20	1	66	13	20/33
	5b	NPV	79	66	13	20	1	
	6a	PPV	7	6	1	58	35	6/41
	6b	NPV	93	58	35	6	1	
	7a	PPV	12	9	3	77	11	9/20
	7b	NPV	88	77	11	9	3	
	8a	PPV	87	60	27	10	3	60/63
	8b	NPV	13	10	3	60	27	
	9a	PPV	65	35	30	21	14	35/49
	9b	NPV	35	21	14	35	30	
	10a	PPV	70	63	7	21	9	63/72
	10b	NPV	30	21	9	63	7	

Correct responses are shown as fractions; proportional equivalents of these fractions were also accepted.

Problem order was randomized within each phase to minimize order effects. Participants were instructed to read each problem carefully before responding, and a minimum response time of 20 s was imposed for each problem. Text copying was disabled to discourage reliance on recall or external assistance.

### 2.3 Procedure

The study was programmed and conducted using Qualtrics (Qualtrics LLC, Provo, UT, USA), a web-based platform for designing and administering online surveys and experiments. The experiment unfolded across three sessions distributed over multiple days, comprising five test phases in total. In session 1, participants completed the pre-test phase, in which they all solved four Bayesian problems without feedback, providing a measure of their baseline performance. One day later (session 2), participants completed the training phase, in which they solved six new problems presented according to their assigned training condition. Immediately after training, they completed a first test (immediate post-test phase), in which they solved the same four problems as in the pre-test, again without feedback, to assess immediate learning effects. Finally, 8–10 days later (session 3), participants completed a post-test (delayed post-test phase) based on the original test problems, followed by a new test (generalization phase) involving problems that required estimation of the predictive value not practiced in the earlier phases (i.e., participants in the PPV track were presented with NPV problems, whereas those in the NPV track were presented with PPV problems).

### 2.4 Training conditions

Participants were exposed to one of three training conditions that differed in the type of support provided. Importantly, participants were not instructed on how to compute Bayesian probabilities, nor were they taught Bayes’ rule or given step-by-step solution strategies. Figure 1 shows an example of how the problems were displayed across the three experimental conditions.

In the *no training condition*, participants were presented with Bayesian inference problems expressed in natural frequencies and solved the tasks without receiving any form of feedback. This served as the control condition, allowing us to assess the extent to which performance improvements could be attributed to repeated exposure to Bayesian problems alone.

In the *textual training condition*, participants solved the same Bayesian inference problems presented in the no training condition, but the textual information was enhanced through visual cues such as color and boldface to highlight key numerical elements (e.g., base rates and test outcomes). In addition, participants received immediate binary feedback after each response indicating whether their answer was correct or incorrect. No explanatory or corrective information was provided. This condition was designed to assess whether the combination of minimal visual emphasis and feedback improves performance beyond mere task exposure.

In the *dynamic training condition*, the textual problem description was accompanied by a 32-s animated graphical representation of the underlying frequency structure (see an example at https://osf.io/gneqv/overview?view_only=591199dde8704f84a2be695017062761), and participants received the same binary feedback as in the textual training condition. The visualization depicted the population as a set of discrete individuals (icons), with different shapes indicating disease status and different colors indicating test outcomes, and illustrated the sequential process by which subsets were generated. The design of the animation made the nested-set structure of the problem visually explicit and was guided by principles derived from the Cognitive Theory of Multimedia Learning (Mayer, 2005) and Gestalt psychology. In particular, *signaling* was implemented through the highlighting of key numerical and verbal information, *spatial contiguity* was ensured by placing text in proximity to the corresponding visual elements, and information was disclosed progressively to reduce cognitive load. In addition, *grouping principles* such as proximity, similarity, and common fate were used to reinforce the perception of set membership and transitions between subsets. Participants were required to view the animation in full at least once before responding. After this initial exposure, they were allowed to replay, pause, or slow down the animation as needed. As in the textual training condition, feedback was limited to a binary indication of response accuracy (“correct” vs. “incorrect”), without any detailed explanation or corrective guidance.

The comparison among the three conditions above allowed a stepwise evaluation of increasingly supportive training formats. Specifically, comparing the no training and textual training conditions enabled us to assess whether minimal textual cues and correctness feedback improved performance, whereas comparing the textual and the dynamic training conditions allowed us to determine whether adding a dynamic visual representation yielded benefits beyond those afforded by the same textual support. Finally, comparing the dynamic training and no training conditions allowed us to assess the effect of the complete training. Accordingly, the design does not isolate the effect of feedback *per se*, but it does allow us to examine whether progressively richer forms of brief and lightweight training yield measurable benefits.

### 2.5 Dependent measures

The primary dependent variable was participants’ accuracy on an open-ended probability question, presented in a natural-frequency format. The question targeted either PPV (“Among those who receive a positive test result, how many actually have Disease X?”) or NPV (“Among those who receive a negative test result, how many actually do not have Disease X?”). Participants responded by entering a numerator and a denominator in two blank fields displayed in the format ‘____ out of ____’. This format allowed accuracy to be defined in terms of the correct ratio rather than a single numerical representation. Specifically, responses were scored as correct if the ratio between numerator and denominator corresponded to the correct probability value, regardless of the numbers entered.

In addition, a descriptive classification of non-Bayesian responses was conducted to examine whether error patterns differed as a function of whether participants were asked to estimate PPV or NPV, based on categories derived from prior research with adult populations (e.g., Gigerenzer and Hoffrage, 1995; Pighin et al., 2017):

1. *base-rate only* (i.e., *p*(*H*) in PPV or *p*(not-*H*) in NPV), which considers only the base rate and completely disregards the evidence (for example, “10 out of 100” in Problem 1a);
2. *test characteristics* (i.e., *p*(*E*|*H*) in PPV or *p*(not-*E*|not-*H*) in NPV), which represents how often the evidence occurs when the hypothesis is true (for example, “8 out of 10” in Problem 1a);
3. *evidence-only* (i.e., *p*(*E*) in PPV or *p*(not-*E*) in NPV), which focuses on the occurrence of the evidence among all cases (for example, “17 out of 100” in Problem 1a);
4. *joint occurrence* (i.e., *p*(*H*&*E*) in PPV or *p*(not-*H*&not-*E*) in NPV), which indicates how often both the hypothesis and the evidence occur among all cases (for example, “8 out of 100” in Problem 1a).

We introduced an additional category, *opposite predictive value*, to capture responses in which participants provided the predictive value opposite to the one requested, suggesting confusion between PPV and NPV (such errors are especially plausible in the generalization phase). Incorrect answers that did not fall into any of these categories were classified as *other* (e.g., “10 out of 517,” “20 out of 1,000,” or “50 out of 993” in Problem 1a).

## 3 Results

Accuracy rates (see Table 2) show a general improvement across phases in both predictive-value conditions: the proportions of correct responses increased from the pre-test to the immediate post-test, and were maintained at delayed post-test and generalization, suggesting that participants benefited from task exposure and practice and that the gains achieved with the interventions were sustained over time. A similar pattern emerged for both PPV and NPV, suggesting that the observed gains generalized across predictive-value conditions. Taken together, these findings point to a robust learning effect across phases, independent of training.

**Table 2:** Accuracy rate across experimental phases as a function of the predictive-value condition and training condition.

PV	Training condition	Pre-test	Training	Immediate post-test	Delayed post-test	Generalization
PPV	No training	34.9	54.4	59.9	58.5	59.4
	Textual training	38.6	60.0	65.7	64.3	54.3
	Dynamic training	48.8	67.9	70.8	65.5	64.9
NPV	No training	43.1	61.6	73.6	67.4	64.6
	Textual training	31.5	57.2	64.1	54.3	59.2
	Dynamic training	39.8	52.3	54.0	54.0	61.9

**Figure 2:**
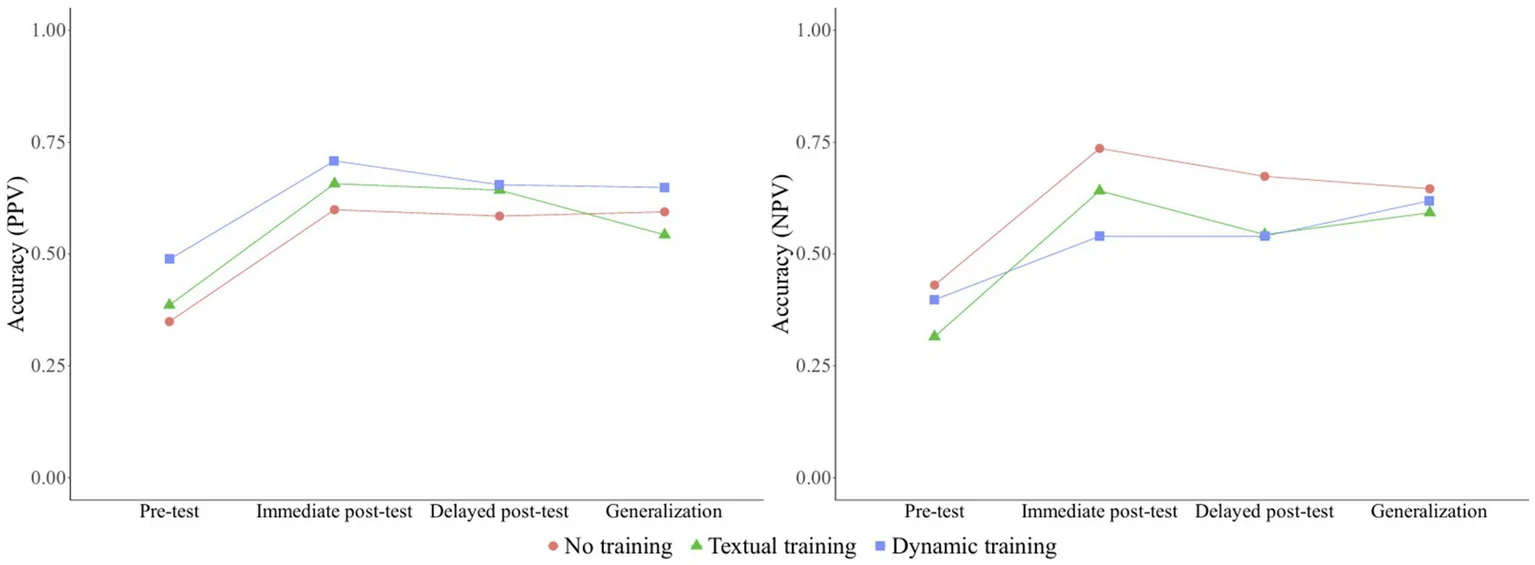
Predicted accuracy across phases as estimated by the GLMM, separately for the PPV track (left panel) and NPV track (right panel), in the three training conditions: no training, textual training, and dynamic training.

This pattern was confirmed by a generalized linear mixed model (GLMM), which included test phase (pre-test, immediate post-test, delayed post-test, generalization), predictive-value condition (PPV vs. NPV), and training condition (no training, textual training, dynamic training), and their interactions as fixed effects. Participant IDs were included as random intercepts. The model (see Figure 2) revealed a significant main effect of phase, *χ*^2^(3) = 329.11, *p* < 0.001, indicating that accuracy varied reliably across phases. Relative to the pre-test, accuracy was significantly higher in the immediate post-test, *b* = 3.983, *SE* = 0.249, *z* = 16.03, *p* < 0.001, *OR* = 53.70, 95% *CI* [32.99, 87.40], in the delayed post-test, *b* = 3.089, *SE* = 0.215, *z* = 14.36, *p* < 0.001, *OR* = 21.97, 95% *CI* [14.41, 33.49], and in the generalization, *b* = 2.988, *SE* = 0.206, *z* = 14.51, *p* < 0.001, *OR* = 19.84, 95% *CI* [13.25, 29.71]. Thus, accuracy increased markedly after the pre-test, and this improvement was maintained over time and extended to the unpracticed predictive value. No significant main effect emerged for training condition, *χ*^2^(2) = 0.57, *p* = 0.753, or predictive-value condition, *χ*^2^(1) = 0.03, *p* = 0.860. The omnibus tests also indicated significant interactions between phase and predictive-value condition, *χ*^2^(3) = 8.85, *p* = 0.031, phase and training condition, *χ*^2^(6) = 18.17, *p* = 0.006, and the three-way interaction among phase, predictive-value condition, and training condition, *χ*^2^(6) = 27.27, *p* < 0.001. However, inspection of the parameter estimates and the descriptive trajectories did not reveal a consistent or theoretically interpretable pattern indicating that either textual or dynamic training produced a systematic advantage over repeated exposure alone. In particular, the significant interaction terms did not correspond to a stable benefit of the training conditions across predictive-value condition and test phase. We therefore interpret the results as evidence for a robust general improvement across repeated testing, with no reliable support for an added benefit of the lightweight training interventions.^1^

Inspection of errors (see Table 3) showed that, across phases, no substantial proportion could be readily mapped onto the standard non-Bayesian categories and therefore had to be classified as *other*. This suggests that many incorrect responses did not conform to any of the reasoning strategies identified in the literature. Among the classifiable errors, responses based on *test characteristics* and *base rates* were the most prominent. Errors involving *test characteristics* were especially frequent in the NPV condition, whereas *base-rate only* responses occurred less consistently. Together, these errors suggest that participants often relied on salient task parameters in isolation, rather than integrating the relevant quantities according to Bayesian reasoning. *Evidence-only* and *joint-occurrence* errors were less frequent and showed no clear temporal pattern. Finally, responses classified as the *opposite predictive value* were virtually absent, even in the generalization phase. This suggests that participants generally attended to the task demands and were able to distinguish which predictive value had to be estimated, even when their responses were incorrect.

**Table 3:** Percentage of error types across phases, by predictive-value condition (PPV vs. NPV).

Type of error	Condition							
	PPV				NPV			
	Pre-test	Immediate post-test	Delayed post-test	Generalization	Pre-test	Immediate post-test	Delayed post-test	Generalization
Base rate only	14	13	11	1	11	5	9	14
Test characteristics	15	14	13	4	35	33	26	26
Evidence-only	11	14	13	20	8	12	6	14
Joint occurrence	14	7	12	11	11	12	6	6
Opposite PV	0	0	0	6	0	0	0	2
Other	46	52	51	58	35	38	53	38

Percentages are calculated over the total number of incorrect responses in each phase.

## 4 Discussion

The present study investigated whether two lightweight training interventions could enhance Bayesian reasoning beyond the effects of mere task repetition. These interventions were compared within an experimental framework designed to assess short-term performance, retention over time, and generalization from one predictive value to the other on both positive and negative predictive value problems. The findings indicate a clear and robust effect of practice: accuracy improved from pre-test to immediate post-test, remained elevated relative to pre-test, and extended to the generalization phase. Importantly, the statistically significant interactions did not translate into a consistent advantage for either training condition over repeated exposure alone. Performance was also broadly comparable across PPV and NPV tasks. Overall, these results suggest that repeated exposure alone can substantially improve both types of judgments and support their generalization.

The potential role of dynamic visualizations in Bayesian training deserves separate consideration. Based on previous work on multimedia learning and visual reasoning (Mayer, 2002, 2005; Han et al., 2012; Zikmund-Fisher et al., 2012), we expected that an animated representation of the nested-set structure would facilitate the integration of statistical information. However, the lack of support for this prediction should not be taken to imply that dynamic visualizations are generally ineffective. Rather, their impact may depend on the broader representational context in which they are introduced (see also Cui et al., 2023). To avoid floor effects and to fully exploit the advantages of a sequential dynamic visualization, all problems were presented in a natural-frequency format, which is known to facilitate Bayesian reasoning by making the nested relations among the relevant sets transparent and enabling the target posterior probability to be derived directly from counts (Barbey and Sloman, 2007; Girotto and Gonzalez, 2001). However, the absence of differences between training conditions is unlikely to be primarily attributable to a ceiling effect, not only because initial accuracy never exceeded 50%, but also because the same pattern observed in the full sample emerged when the analyses were restricted to participants who answered none of the pre-test problems correctly and therefore had the greatest scope for improvement. Nevertheless, the present findings should not be assumed to generalize to standard probability formats, in which base rates and test characteristics are expressed relative to different reference classes and must be integrated into a common representation to compute posterior probability. When information is presented in this more demanding format, a dynamic visualization that explicitly supports the construction of an alternative representation may provide an advantage beyond repeated exposure. Future studies could investigate this issue by comparing numerical formats (i.e., natural frequencies vs. single-case probabilities) while applying the same dynamic intervention to both, to determine the extent to which the pattern observed here is specific to the problem representation used.

A relevant contribution of the present study lies in its direct comparison of PPV and NPV reasoning using matched problems with identical computational complexity. To our knowledge, this is the first study to consider this kind of controlled comparison. As noted in the Introduction, the literature has focused on PPV, and the few studies that have also examined NPV have not always yielded findings comparable to those reported for PPV. When potential confounds were controlled for, as in the present study, no reliable difference in accuracy emerged between the two predictive values. This suggests that, when inferential demands are carefully equated and information is presented in a facilitative format, PPV and NPV may be similar in difficulty. At the same time, descriptive differences in error patterns indicate that comparable levels of accuracy do not necessarily reflect identical reasoning processes, as *test-characteristics* errors were more frequent in the NPV condition. Although these findings remain descriptive at this stage, they underscore the importance of distinguishing between performance outcomes and the inferential processes elicited by the two predictive-value tasks.

The error analysis further qualifies the interpretation of the accuracy findings. A substantial proportion of incorrect responses fell into the *other* category, suggesting that they were too heterogeneous to be adequately captured by the standard non-Bayesian response patterns described in previous research (Gigerenzer and Hoffrage, 1995; Pighin et al., 2017; Pighin et al., 2024). This may, in turn, suggest that incorrect responses often reflected weakly structured reasoning attempts, rather than the systematic use of a limited set of identifiable alternative strategies. Among the classifiable errors, responses based on *test characteristics* or *base rates* were the most frequent, suggesting that participants often relied on salient task parameters without fully integrating the nested-set relations required for Bayesian inference. These two error types also seem to reflect opposite failures of updating: in the former case, participants rely primarily on the diagnostic evidence explicitly provided in the problem (e.g., sensitivity or specificity), as though it directly answered the inferential question, thereby neglecting the base rate; in the latter, they rely mainly on the prior probability alone, as though the test result conveyed no additional information.

The findings of this study should be interpreted in light of some limitations. First, our study deliberately focused on a relatively simple Bayesian problem. Accordingly, all problems were presented in a nested natural-frequency format, a choice motivated by the need to reduce floor effects and to make performance changes across phases and training conditions easier to detect. We adopted this format because, even when Bayesian problems are presented in this way, Bayesian reasoning remains challenging, likely because the main difficulty lies not only in carrying out the required computation, but also in constructing and integrating an appropriate representation of the information (see Pighin et al., 2024). As noted above, however, this choice may also have reduced the opportunity for dynamic visualization to yield an additional benefit. A similar consideration applies to the training procedure, which was intentionally designed to be lightweight: participants were not taught Bayes’ rule, were not given explicit solution strategies, and received only minimal support. Although this design enhanced the ecological plausibility and practical applicability of the interventions, it may also have limited their capacity to produce a robust performance advantage.

Second, the immediate post-test, delayed post-test, and generalization phases presented the same numerical values as those used in the pre-test phase. This choice allowed performance to be compared across phases under highly controlled conditions, minimizing variability due to changes in problem content or numerical values, thereby facilitating interpretation of performance changes. At the same time, however, this does not rule out the possibility that at least part of the observed improvement reflected increased familiarity with the inferential structure of the problems, rather than a deeper acquisition of transferable Bayesian reasoning skills. Although participants were unlikely to remember the specific numerical values after a delay of 8–10 days, they may nevertheless have retained the underlying problem structure.

Third, our training interventions combined multiple components: minimal textual cueing and binary feedback in one condition, and dynamic representations and feedback in the other. Future research could enhance these components (e.g., by providing more informative feedback) and examine their individual contributions using less facilitative numerical formats, alternative problem sets, and process-sensitive measures better suited to capturing participants’ reasoning pathways.

In conclusion, the present study suggests that repeated exposure to Bayesian problems in a supportive representational format is sufficient to yield modest but durable improvements, together with some degree of transfer. From an applied perspective, this is encouraging, as it suggests that even relatively low-cost interventions may promote better probabilistic reasoning in contexts where full-scale training is impractical. At the same time, further research is needed to determine the conditions under which dynamic visualizations offer added value beyond practice, minimal feedback, and format-based facilitation.

## Data Availability

The datasets presented in this study can be found in online repositories. The names of the repository/repositories and accession number(s) can be found at: https://osf.io/gneqv/overview?view_only=591199dde8704f84a2be695017062761.

## Footnotes

1 The same overall pattern of results emerged when analyses were restricted to the subset of participants with the poorest pre-test performance (*n* = 129), namely those who answered none of the four pre-test problems correctly. Even within this subgroup, no reliable differences were observed across training conditions. Thus, neither training appeared to offer a substantial advantage over mere repetition, even for participants who seemed most in need of support.
